# A randomised controlled trial investigating the clinical and cost-effectiveness of Alpha-Stim AID cranial electrotherapy stimulation (CES) in patients seeking treatment for moderate severity depression in primary care (Alpha-Stim-D Trial)

**DOI:** 10.1186/s13063-022-06192-1

**Published:** 2022-04-04

**Authors:** Shireen Patel, Clement Boutry, Priya Patel, Michael P. Craven, Boliang Guo, Azhar Zafar, Joe Kai, David Smart, Debbie Butler, Fred Higton, Rebecca McNaughton, Paul M. Briley, Chris Griffiths, Neil Nixon, Kapil Sayal, Richard Morriss

**Affiliations:** 1 Institute of Mental Health, University of Nottingham, Nottingham, UK; 2 NIHR Applied Research Collaboration East Midlands, Leicester, UK; 3 NIHR Nottingham Biomedical Research Centre, Nottingham, UK; 4 NIHR MindTech MedTech Co-operative, Nottingham, UK; 5 General Practice Alliance (GPA) Federation Northants, Northampton, UK; 6 Leicester Diabetes Centre University of Leicester, Leicester, UK; 7 Centre for Academic Primary Care, School of Medicine, University of Nottingham, Nottingham, UK; 8 NIHR School for Primary Care Research, University of Nottingham, Nottingham, UK; 9 Nottinghamshire Healthcare NHS Foundation Trust, Nottingham, UK; 10 Northamptonshire Healthcare NHS Foundation Trust, Kettering, UK

**Keywords:** Depression, Primary care, Alpha-Stim AID, Cranial electrotherapy stimulation, Antidepressants, Randomised controlled trial

## Abstract

**Background:**

Major depression is the second leading cause of years lost to disability worldwide and is a leading contributor to suicide. However, first-line antidepressants are only fully effective for 33%, and only 40% of those offered psychological treatment attend for two sessions or more. Views gained from patients and primary care professionals are that greater treatment uptake might be achieved if people with depression could be offered alternative and more accessible treatment options. Although there is evidence that the Alpha-Stim Anxiety Insomnia and Depression (AID) device is safe and effective for anxiety and depression symptoms in people with anxiety disorders, there is much less evidence of efficacy in major depression without anxiety. This study investigates the effectiveness of the Alpha-Stim AID device, a cranial electrotherapy stimulation (CES) treatment that people can safely use independently at home. The device provides CES which has been shown to increase alpha oscillatory brain activity, associated with relaxation.

**Methods:**

The aim of this study is to investigate the clinical and cost-effectiveness of Alpha-Stim AID in treatment-seeking patients (aged 16 years upwards) with moderate to moderately severe depressive symptoms in primary care. The study is a multi-centre parallel-group, double-blind, non-commercial, randomised controlled superiority trial. The primary objective of the study is to examine the clinical efficacy of active daily use of 8 weeks of Alpha-Stim AID versus sham Alpha-Stim AID on depression symptoms at 16 weeks (8 weeks after the end of treatment) in people with moderate severity depression. The primary outcome is the 17-item Hamilton Depression Rating Scale at 16 weeks. All trial and treatment procedures are carried out remotely using videoconferencing, telephone and postal delivery considering the COVID-19 pandemic restrictions.

**Discussion:**

This study is investigating whether participants using the Alpha-Stim AID device display a reduction in depressive symptoms that can be maintained over 8 weeks post-treatment. The findings will help to determine whether Alpha-Stim AID should be recommended, including being made available in the NHS for patients with depressive symptoms.

**Trial registration:**

ISRTCN ISRCTN11853110. Registered on 14 August 2020

## Administrative information

Note: the numbers in curly brackets in this protocol refer to SPIRIT checklist item numbers. The order of the items has been modified to group similar items (see http://www.equator-network.org/reporting-guidelines/spirit-2013-statement-defining-standard-protocol-items-for-clinical-trials/).

**Table:** 

Title {1}	A randomised controlled trial investigating the clinical and cost-effectiveness of Alpha-Stim AID cranial electrotherapy stimulation (CES) in patients seeking treatment for moderate severity depression in primary care (Alpha-Stim-D Trial)
Trial registration {2a and 2b}	This trial has been registered on the ISRTCN Registry. ISRTCN Number: ISRCTN11853110. Registered on 14/08/2020.
Protocol version {3}	This protocol is version 1.2, dated 04/06/2020
Funding {4}	NIHR Applied Research Collaboration East Midlands (ARC EM), Electromedical Products International, Inc.
Author details {5a}	1. Institute of Mental Health, University of Nottingham 2. NIHR Applied Research Collaboration East Midlands 3. NIHR Nottingham Biomedical Research Centre 4. NIHR MindTech MedTech Co-operative 5. Centre for Academic Primary Care, School of Medicine, University of Nottingham 6. NIHR School for Primary Care Research 7. General Practice Alliance (GPA) Federation Northants 8. Leicester Diabetes Centre University of Leicester 9. Nottinghamshire Healthcare NHS Foundation Trust 10. Northamptonshire Healthcare NHS Foundation Trust
Name and contact information for the trial sponsor {5b}	Ms Angela Shone, University of Nottingham, Research and Innovation University of Nottingham East Atrium Jubilee Conference Centre Triumph Road Nottingham NG8 1DH.
Role of sponsor {5c}	The sponsor and the investigators participating in the trial bear the final responsibility for the conduct of the trial. This study is funded by the National Institute for Health Research (NIHR) Applied Research Collaboration East Midlands (ARC EM). The funder plays no role in the design of the study; collection, analysis and interpretation of the data; and writing. Electromedical Products International, Inc. is providing additional funding to cover excess treatment costs, health economic assessment and supply of devices. The Microcurrent Site enables the allocation of the devices following randomisation and arranges collection of devices. They play no role in the design of the study; collection, analysis and interpretation of the data; and writing.

## Introduction

### Background and rationale {6a}

#### Existing research: prevalence and treatment of depression

Major depression is the second leading cause of years lost to disability in the world [1]. From 2007 to 2014, the United Kingdom National Household Survey showed an escalating demand for the treatment of depression and anxiety (1 in 6 adults [2]). Clinically effective, cost-effective and safe management of common mental illness, such as depression and anxiety, using a variety of approaches in integrated care systems is a priority for the NHS10 year long-term plan, as is addressing inequalities such as those associated with poverty and ethnicity [3]. Almost a quarter of 17–19-year-old women meet the diagnostic criteria for an emotional disorder [4]. According to the Office for National Statistics (ONS), since the beginning of the coronavirus pandemic (COVID-19), the rates of depression in the community have more than doubled; around one in five (21%) aged 16 years and over in Great Britain experienced some form of depression (indicated by moderate to severe depressive symptoms) in early 2021 (27 January to 7 March) [5]. The rates were more than double those observed before the coronavirus (COVID-19) pandemic, when 10% of adults experienced some form of depression [5]. The impact was felt more greatly in younger adults, women and people who live alone—with 43% of women aged between 16 and 29 reporting some form of depression [5].

Currently, the two main forms of treatment offered for depression by the National Health Service (NHS) in the United Kingdom (UK) are antidepressant medications and psychological treatments. However, first-line use of antidepressants is only effective for 1 in 3 of those who try them, and over 1 year, approximately 66% remit. Furthermore, antidepressants are associated with a range of side effects, e.g. sedation, weight gain, cardiac arrhythmia and falls, suggesting that they may not be tolerated by some people, particularly those with physical ill health [6]. Psychological treatments are as effective as antidepressants; however, only 40% of those offered psychological treatments attend for two sessions or more, and of those treated, 49% do not progress or recover [7]. Despite a large increase in the prevalence of depression and evidence of increased mental health burden due to the COVID-19 pandemic, there were significant reductions in contact with primary services in the UK for mental illness from April 2020 [8]. However, subsequently, primary care mental health demands have risen since the early period of the pandemic, and there has been a rapid transition to virtual care [9].

The current increase in depression rates, coupled with challenges in accessing existing psychological treatments, suggests a need for the availability of novel treatments for depression to be offered as part of routine NHS care. Our consultation with patient and public involvement and engagement (PPI/E) representatives and primary care practitioners indicated that novel treatment approaches that were effective, easily accessible and could be used at home with minimal supervision may increase treatment uptake without overburdening primary care services.

#### Cranial electrotherapy stimulation (CES)

CES is an established form of non-invasive low alternating current neuromodulation treatment. It is related to other forms of transcranial electrical stimulation, including electroconvulsive therapy and transcranial direct current stimulation. CES was developed in Russia in 1900 and was first used to induce sleep and relaxation [10] by applying small electric currents to the head. Since the 1970s, CES has been used increasingly in clinical settings. In America, one of the most commonly used CES devices since the 1980s is Alpha-Stim (Electromedical Products International, Inc.). Alpha-Stim AID is the latest model of electrotherapy device for the treatment of anxiety, insomnia and depression. Alpha-Stim AID was CE marked in 2012 as a class IIa medical device. It is a small mobile phone-sized device powered by two AAA batteries and is attached by leads to both earlobes. It delivers a patterned, subsensory (cannot be felt or detected), electrical waveform and has been associated with a reduction in delta (0–3.5 Hz) and beta (12.5–30 Hz) frequency brainstem and cortical electroencephalography (EEG) activity and an increase in alpha (8–12 Hz) activity (associated with states of relaxation) [11]. It can be used daily for 20–60 min. The device with a 5-year maintenance package retails at approximately £500 with a 5-year warranty. However, since depression is associated with social deprivation, the cost of the device is a significant barrier to its uptake for the treatment of depression with the majority of people with depression unable to afford the device [12].

#### Evidence base for Alpha-Stim AID

A systematic review carried out by the US Veterans Administration identified 5 randomised controlled trials (RCTs) with 198 participants for anxiety or mixed anxiety and depression disorders comparing CES to sham CES [13]. The review concluded that there was low-quality evidence of the effectiveness of CES in relation to anxiety and depression symptoms. A previous study reported that Alpha-Stim AID significantly reduced depression from baseline to endpoint of the study [14]. Moreover, in a trial of 115 participants with primary anxiety disorder, 5 weeks of active Alpha-Stim AID 100 was shown to be more effective than sham devices in reducing depression [15]. Recently, researchers in England carried out an implementation study in the Improving Access to Psychological Therapies (IAPT) setting [16]. The findings indicated that, in 161 patients with moderate to severe generalised anxiety disorder (GAD) who had not responded to first-line psychological treatment, the mean PHQ-9 scores of self-rated depression dropped from 16.1 to 11.2 by week 4. Furthermore, the mean PHQ-9 scores dropped to 10.4 at week 6, and by week 12, PHQ-9 scores were at normal levels (8.9) indicating remission of symptoms at week 12, which was maintained to week 24. There was slippage in the mean score of depression by less than 1 point on the PHQ-9 at 24 weeks suggesting that a course of 8 weeks of using Alpha-Stim AID rather than 6 weeks CES may be optimal for the treatment of depression symptoms. There was similar moderate to large improvements in anxiety symptoms. Only four (2.5%) participants withdrew from treatment because of side effects. Compared to individual cognitive behavioural therapy (CBT), Alpha-Stim AID provided a cost saving of £540.88 per patient (95% confidence interval £327.12, £648.69). Cost modelling by the company suggests that the cost of Alpha-Stim AID could be as low as £40 in primary care [16]. However, there are no trials of cost-effectiveness of Alpha-Stim AID for depression in primary care settings. Furthermore, there is a need for further sham-based RCTs, since the daily routine of using a device might in itself have benefits on depression since structuring the day is a technique used in some behaviour therapy treatments for depression [17]. The device might therefore seem to be effective through an active placebo effect.

The NHS in the UK and other health services require more evidence of clinical and cost-effectiveness in a primary care setting, where similar devices are routinely used for other long-term ‘physical health’ conditions and where the majority of treatment for depression takes place [18]. Although the evidence base, tolerability, patient preference and ease of use are promising, a recent report by NICE [18] recommends that there is a need for a further randomised controlled trial of the long-term clinical and cost-effectiveness in treatment-seeking patients with moderate depression in primary care. This research will investigate if offering Alpha-Stim AID to people with moderate depression in primary care can reduce depression, provide more evidence of tolerability and examine cost-effectiveness.

### Objectives {7}

The overall purpose and objective of the trial are to determine the clinical and cost-effectiveness of active Alpha-Stim AID compared to sham Alpha-Stim AID for depressive symptoms in people with moderate to moderately severe depressive symptoms in primary care.

#### Primary objective

The primary objective of the study is to explore the clinical efficacy of active Alpha-Stim AID versus sham Alpha-Stim AID on depressive symptoms at 16 weeks (8 weeks after the end of treatment) in people with moderate to moderately severe depressive symptoms. This will be measured using the Grid version of the Hamilton Depression Scale (GRID-HAMD [19]).

#### Secondary objectives

The following are the secondary objectives:

1. To evaluate the cost-effectiveness of Alpha-Stim AID compared to sham Alpha-Stim AID. This will be analysed through the measurement of costs from personal, health and social care perspectives using Client Service Receipt Inventory (CSRI) [20] at 4, 8 and 16 weeks. Quality-adjusted life years (QALYs) will be estimated using the EQ-5D-5L [21].
2. To evaluate the effectiveness of secondary outcomes deemed important by clinicians and patients. To measure this, the following outcome measures will be used:
  - GRID-HAMD at 4 and 8 weeks
  - Nine-item Patient Health Questionnaire for depression (PHQ-9 [22];) at 4, 8 and 16 weeks
  - Seven-item Generalised Anxiety Disorder (GAD-7 at 4, 8 and 16 weeks [23]
  - Functioning using the 8-item Work and Social Adjustment Scale (WSAS [24];) at 4,8 and 16 weeks

### Trial design {8}

The study is a multi-centre parallel-group, double-blind, intention-to-treat superiority RCT. The study will be undertaken in primary care general practice (GP) practices for people with moderate to moderately severe depressive symptoms. Participants will be randomised in a 1:1 ratio to 8 weeks of treatment of either active Alpha-Stim AID or sham Alpha-Stim AID. Randomisation will be conducted online via a system set up by the University of Nottingham’s (UoN) Clinical Database Support Service (CDSS) with blinding of participants and researchers collecting the outcome measures. Participants in both arms will receive the same outcome measures. Participants will be followed up at mid-treatment (4 weeks), end treatment (8 weeks) and 8 weeks post-intervention (16 weeks).

## Methods: participants, interventions and outcomes

### Study setting {9}

**Fig. 1:**
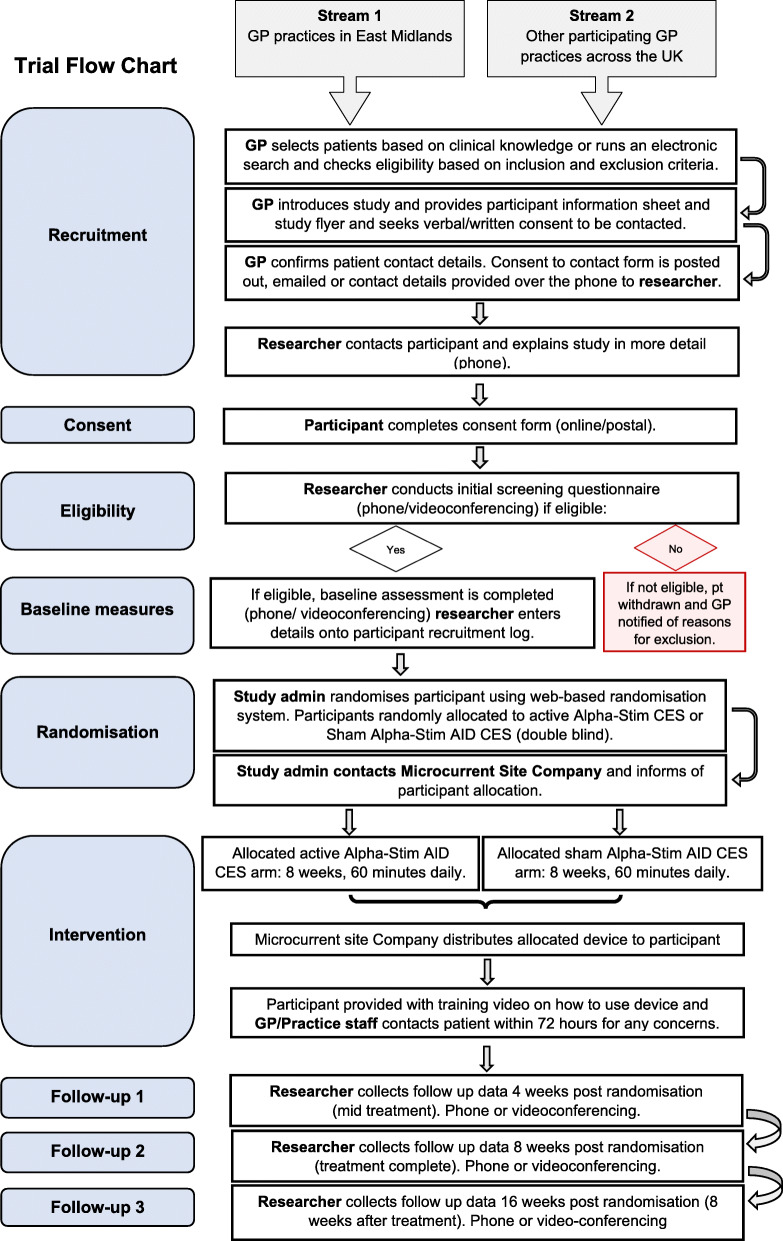
Trial flow chart

The study is a multicentre study based on primary care GP practices in three regions of England, the East Midlands and the Thames Valley and South Midlands. This may extend to additional sites in other regions in England. GP practices are primarily identified via the NIHR Clinical Research Network (CRN). The study is promoted on their website, and GP surgeries expressing an interest are contacted. Practices include a range reflecting differing registered patient list size, patients of differing social and ethnic diversity and affluence or deprivation. The initial approach is from a member of the patients’ usual clinical care team in primary care during routine appointments (face-to-face, over the telephone or video consultation) or via a practice database search and mail out. Information about the trial is also displayed in relevant clinical areas. Participants may also directly contact the research team to be screened to participate in the study, but the appropriateness of their participation is checked with a clinician from their GP surgery to ensure they meet the eligibility criteria. The study flow chart (Fig. 1) provides an overview of the study design and procedures.

### Eligibility criteria {10}

Participants are selected by their primary care clinicians based on the following criteria.

#### Inclusion criteria

The following are the inclusion criteria:

- Aged 16 years and above. There is no maximum age limit.
- Diagnosis of current major depressive episode (MDE). This is confirmed using the research version of the Structured Clinical Interview for DSM-5 (SCID-5-RV [25];) diagnostic clinical interview at baseline.
- A score of ≥ 10 on the 9-item self-rated Personal Health Questionnaire (PHQ-9 [22];).
- Have either been offered the option of antidepressant medication or have been prescribed antidepressant medication for a minimum of 6 weeks in the last 3 months.
- Capable of giving oral and written informed consent to the study.
- Agrees to return Alpha-Stim equipment at the end of the study and not to purchase this equipment privately during the study.

#### Exclusion criteria

The following are the exclusion criteria:

- A score of ≥ 20 on the PHQ-9.
- Neurological conditions, e.g. brain neoplasm, cerebrovascular events, epilepsy, neurodegenerative disorders and prior brain surgery.
- Requires urgent clinical care such as having persistent suicidal ideation, self-harm or suicidal intent.
- Known to be pregnant. Pregnancy is an exclusion criterion and reportable as a precautionary measure as research has not been undertaken on the safety and efficacy of Alpha-Stim AID treatment in pregnant patients.
- Implantation with a pacemaker, cochlear implant or an implantable cardioverter device (ICD).
- Major unstable medical illness requiring further investigation or treatment.
- A diagnosis of current substance use disorder or dependence, dementia, eating disorder, bipolar disorder or non-affective psychosis because the use of the treatment would require additional supervision or is associated with additional risk, e.g. of mania in bipolar disorder. Determination of these conditions will be confirmed using the SCID-5-RV.
- Completed and benefitted from/responded to psychological treatment for depression in the last 3 months or planning to commence psychological treatment in the next 6 months.
- Involved with any other depression-related clinical trial at the time of consent or 6 months prior.

Being on medication; having a comorbid anxiety disorder, neurodevelopmental disorder, or personality disorder; or having a stable physical illness not requiring urgent clinical care are not exclusion criteria.

### Who will take informed consent? {26a}

Referring clinicians approach patients who meet the eligibility criteria and seek consent to be contacted by the study researchers. Patients who provide written or verbal consent to be contacted by the research team are then telephoned by a researcher. Details about the study and a participation information sheet (PIS) are provided, and informed consent is sought. All participants have at least 24 h from receiving the initial study information to signing consent. The informed consent form is signed and dated by the participant before they enter the trial. Trained, suitably qualified and experienced researchers under the supervision of clinically trained investigators will obtain informed consent and undertake all assessments. A screening questionnaire will be used to telephone screen interested participants first, with potentially eligible participants invited to complete a baseline assessment. Should there be any subsequent amendment to the final protocol, which might affect a participant’s participation in the trial, continuing consent will be obtained using an amended informed consent form, approved by the Research Ethics Committee (REC) and the sponsor, which will be signed by the participant.

### Additional consent provisions for collection and use of participant data and biological specimens {26b}

On the consent form, participants will be asked if they agree to the use of their data should they choose to withdraw from the trial. Participants will also be asked for permission for the research team to share relevant data with authorised individuals from the University of Nottingham, the research group and regulatory authorities where relevant. This trial does not involve collecting biological specimens for storage.

### Interventions

#### Explanation for the choice of comparators {6b}

The active device used in this study will be the CE-marked Alpha-Stim AID 100. The sham Alpha-Stim AID device will be identical to the active device, looking and sounding the same and clearly switched on but the ear clip electrodes do not emit electricity. The Alpha-Stim AID 100 device electrodes emit electricity at a subsensory threshold which is associated with very low rates of side effects (2.5% or less).

#### Intervention description {11a}

The active device used in this study is the CE-marked Alpha-Stim AID 100 manufactured by Electromedical Products International, Inc. The device provides electrical stimulation by generating bipolar, asymmetric, rectangular waves with a frequency of 0.5 Hz and a current intensity that is pre-set and locked by the manufacturer at its lowest therapeutic dose at 100 μA, a subsensory level. This level has been found to be effective [15, 26]. The unlocked device is marketed and sold in the UK available from private purchase at a cost of approximately £500.

Participants will be informed that they are expected to undertake 60-min daily self-directed Alpha-Stim AID treatment sessions for 8 weeks. Participants will receive an instructional video on the use of the device and a written leaflet, and they will be advised to contact the practice staff or study team if they have any queries. Participants will be provided with treatment logs to document the day, time and duration of treatment. A GP, nurse or health care assistant (HCA) from the GP surgery will telephone each participant within 72 h of starting active Alpha-Stim AID or sham Alpha-Stim AID treatment to discuss any uncertainties, record and discuss side effects and check participants’ mental state to ensure that participants are not at risk of self-harm or suicide. If there are any concerns related to the participant’s safety, then the GP and research team will be informed.

The sham Alpha-Stim AID devices will be identical to the active device in look, sound and feel, but the ear clip electrodes will not emit electricity. Participants who receive the sham device will be provided with identical information and instructions. They will also receive a 72 h follow-up call. Participants will not be able to change any of the device settings that regulate current, frequency and time on either of the devices. The manufacturers (Electromedical Products International, Inc.) will test the active and sham devices before they will be shipped to the distributors in the UK (the Microcurrent Site Ltd.) to ensure that sham devices are not emitting a current.

#### Criteria for discontinuing or modifying allocated interventions {11b}

Data on the impact of the intervention on reducing depressive symptoms and other outcome measures will not be analysed until the end of the study period and therefore will not inform decisions to stop the research. However, serious adverse events (SAEs) and serious adverse device events (SADEs) will be reviewed, and if there is any indication that these are linked to the intervention, consideration will be given to stopping on the advice of the National Institute for Health Research (NIHR) Applied Research Collaboration (ARC) East Midlands (EM) Scientific Committee and study sponsor.

#### Strategies to improve adherence to interventions {11c}

Participants will be provided with treatment logs to document the day, time and duration of treatment. Adherence will be determined from self-report and use of the machine (calculated from the counter that is part of the display output of the machine). This gives the total amount of time used. We will consider participants to be adherent if they have used the machine for a minimum of 28 h, i.e. at least 50% of the intervention time. This is because in a previous study, 75% of improvement in depression symptoms with Alpha-Stim AID occurred in the first 4 weeks of the intervention [16].

#### Relevant concomitant care permitted or prohibited during the trial {11d}

Participants will be advised to return the device within a maximum of 10 weeks from receipt of the device. This will be stipulated in the participation information sheet and consent form. We will advise participants not to purchase the device privately during their participation in the study, but they may wish to purchase it thereafter. If participants inform the researchers that they have purchased a device during their participation in the study, this will be recorded on the CSRI. Participants who are already on any antidepressant or anti-anxiety medication will be asked to remain on the same dose of their medication during their participation in the study. Participants will also be requested not to commence any structured psychological therapy during their participation in the trial.

#### Provisions for post-trial care {30}

Following participation in the study, participants can continue to seek help for their depression with their usual treating clinicians. They will not have access to the Alpha-Stim AID device free of charge through the NHS after the trial.

### Outcomes {12}

The primary clinical outcome is change on the 17-item GRID version of the Hamilton Depression Scale (GRID-HAMD [19];) from baseline to 16 weeks.

One of the secondary clinical outcomes is change from baseline to 16 weeks on the following:

- Nine-item Patient Health Questionnaire for depression (PHQ-9 [22];)
- Seven-item generalised anxiety disorder scale (GAD-7 [23];)
- Eight-item work and social adjustment scale (WSAS [24];)
- Five-item quality of life on the EQ-5D-5L [21]
- Change in healthcare service use established through an adapted version of the CSRI [20];)

#### Participant timeline {13}

Participants who are eligible and consent to participate in the trial have one screening/baseline assessment (which is over the telephone or via videoconferencing). In addition, they have three follow-up assessments at weeks 4, 8 and 16. Study processes are undertaken by the staff in GP practices and trial researchers, in addition to staff from NIHR Clinical Research Network (CRN).

The following measures will be collected from the participants in both trial arms:

1. GRID-HAMD [19]
2. PHQ-9 [22]
3. GAD-7 [23]
4. WSAS [24]
5. EQ-5D-5L [21]
6. CSRI [20]

All measures are self-rated but will be administered over the telephone or via video-conferencing with a study researcher. Some items on the GRID-HAMD are rated by the researcher. The participant is also interviewed using the research version of the Structured Clinical Interview for Diagnostic and Statistical Manual of Mental Disorders (SCID-5-RV [25];) psychiatric interview. This records the absence or presence of depressive disorders, anxiety disorders, bipolar disorders, eating disorders and substance use disorders.

Demographic information and SCID-5-RV is collected at baseline/screening assessment only. Demographic information will consist of questions about ethnicity, education and employment. The other measures are collected at baseline/screening and 4, 8 and 16 weeks post-randomisation.

Table 1 outlines the completion time points for each measure and who completes them.

**Table 1:** Baseline/screening and outcome measures

Time point	0	0	1	2	4	Completed by
Months post randomisation	Pre-baseline/screening	Baseline	Mid-treatment (4 weeks)	Complete-treatment (8 weeks)	8 weeks after completion (16 weeks)	
**Consent**	X					P
**GAD7**		X	X	X	X	R and P
**WSAS**		X	X	X	X	R and P
**EQ-5D-5L**		X	X	X	X	R and P
**CSRI**		X	X	X	X	R and P
**SCID-5-RV**	X (major depressive episode criteria)	X				R and P
**GRID-HAMD**		X	X	X	X (primary outcome)	R and P
**Randomisation**		X				R
**PHQ-9**	X		X	X	X	R and P

*R* researcher, *P* participant

#### Sample size {14}

A between-group difference of 3 points on the GRID-HAMD scale at follow-up is an internationally accepted minimum clinically important difference for depression disorders (NICE, 2004). With reference to the results shown in an RCT of a similar population [27], a sample size of 86 per arm will be required to detect a between-group mean difference of 3 points (SD = 6.4) on GRID-HAMD at 16 weeks follow-up with 90% power and two-tailed significance of 5%, assuming a correlation between baseline and follow-up measure of 0.1 and between follow-up measure correlation of 0.85. To allow for up to 25% missing primary outcome data, we plan to recruit 230 participants (115 per arm). Stata sampsi code was used to calculate the power.

#### Recruitment {15}

We are adopting various strategies to ensure adequate participant recruitment. This has involved utilising opportunistic and mail out (via Docmail) referral approaches and utilising the support of NIHR’s CRN to identify GP practices.

### Assignment of interventions: allocation

#### Sequence generation {16a}

Randomisation will be conducted via a secure web-based randomisation system. Participants are individually randomised in a 1:1 ratio to active Alpha-Stim AID or sham Alpha-Stim AID, minimised by region, presence of anxiety disorders and use of antidepressant medication (offered versus prescribed).

#### Concealment mechanism {16b}

Participants, researchers rating outcome meausures, GP primary care staff and the trial statistician will be blinded to treatment allocation. The randomisation system will ensure that researchers collating the outcome measures remain blind to the intervention arm. All participants receive the same outcome measures, so that assessors do not know which group the participant is in. The active and sham devices are identical in appearance, sound and feel. Both will be clearly switched on, but the active device will emit current at a subsensory level, and the sham device will not emit any current. Researchers will stress to participants in both arms that they may not be able to feel anything but to be assured that the device is working.

#### Implementation {16c}

Randomisation is conducted via a web-based randomisation system (University of Nottingham’s CDSS) by a study researcher. Study researchers receive a blinded confirmation of randomisation. The study administrator or another member of the study team at the University of Nottingham (not involved in the assessment of the outcome of the participant) will receive an un-blinded randomisation confirmation. They will convey device allocation directly to the Microcurrent Site Ltd. who are provided with the name and address of participants (but are blinded to any baseline clinical or demographic information about the participants). The company will arrange for the distribution of the allocated device to the participant and keep a log of the serial numbers for devices allocated.

### Assignment of interventions: blinding

#### Who will be blinded {17a}

Participants, study researchers and primary care staff will be blinded to treatment allocation.

#### Procedure for unblinding if needed {17b}

Unblinding will only be permissible in the event of a medical emergency or SAEs or SADEs. The study administrator will identify the allocated treatment of participants through password-protected access and convey this information to the chief investigator (CI).

### Data collection and management

#### Plans for assessment and collection of outcomes {18a}

Data will initially be collected on electronic-based source documents. Data will then be transferred onto the REDCap database platform. Data entry and management will be completed by the study researchers, and the sponsor will not have access to the data. All source documents and data sheets used in the trial will not have patient identifiable information; instead, participants will be assigned a participant identifying code. All information pertaining to the study will be retained for 7 years in accordance with the University of Nottingham’s Research Code of Conduct.

#### Plans to promote participant retention and complete follow-up {18b}

The data of all participants including those who discontinue using the device will be collected according to the study protocol. To promote participant retention, 1 week before the follow-up is due, participants will receive a phone call or text message from the researcher. A time will be arranged for the completion of outcome measures. If a response is not received from the participant within 7 days, a reminder call/text will be sent out. An additional two reminders will be sent out. Participants will not be accepted as lost to follow-up unless phone calls/text messages on at least four occasions have been fruitless. If a participant chooses to withdraw from the study, the data collected up to the withdrawal date will be used.

#### Data management {19}

Data will be collected and stored on the REDCap platform. Entries on case report forms (CRFs) will be verified by inspection against the source data. A sample of CRFs (10%) will be checked for verification of all entries made. Where corrections are required, these will carry a full audit trail and justification.

#### Confidentiality {27}

Individual participant information obtained as a result of this study are considered confidential, and disclosure to third parties is prohibited with the exceptions noted above. Participant confidentiality will be further ensured by utilising identification code numbers to correspond to treatment data in the computer files.

If information is disclosed during the study that could pose a risk of harm to the participant or others, the researcher will discuss this with the CI and where appropriate report accordingly to the participant’s primary care clinician. This is highlighted in the participant information sheet. Data generated as a result of this trial will be available for inspection on request by the participating physicians, the University of Nottingham representatives, the Research Ethics Committee (REC), local Research and Development (R&D) Departments and the regulatory authorities.

#### Plans for collection, laboratory evaluation and storage of biological specimens for genetic or molecular analysis in this trial/future use {33}

Please see item 26b; this trial does not involve collecting biological specimens

## Statistical methods

### Statistical methods for primary and secondary outcomes {20a}

The analysis will be conducted on an intention-to-treat (ITT) basis. Exploratory analysis for both primary and secondary outcomes will be conducted first. A detailed trial Statistics Analysis Plan (SAP) setting out full details of the proposed analyses will be finalised before the trial database is locked for final analysis. All the trial data will be stored on UoN secure platforms and analysed on a secure UoN computer. The latest available version of STATA software will be used for all data analysis.

#### Primary outcome analysis

The primary outcome is GRID-HAMD at 16 weeks post-randomisation. The treatment effects on GRID-HAMD score would be quantified using the ANCOVA approach by means of multilevel modelling (MLM) with patients as level 2 analytical unit, arm (active versus sham), follow-up time (discrete variable) and their interaction, together with baseline measure and minimisation factors included as fixed effects covariate. The treatment differences at every follow-up time together with their 95% confidence intervals will be derived from the MLM. As patients will be recruited from various sites, centre details (GP practices), the centre will be included as a higher-level analytical unit if needed after data exploratory analysis.

#### Secondary outcome analysis

A similar analytic approach will be used to quantify the treatment effects on all secondary outcomes. Any skewed outcome variables will be transformed for multilevel modelling.

### Economic evaluation

A within-trial economic evaluation will be undertaken from a health and social care perspective in the base case, with a broader societal perspective considered in secondary analyses in order that impacts on the family, work and benefits are captured in addition to health and social care impacts. Primary care staff will complete bespoke forms to record their time dedicated to the intervention; these staff intervention costs will be estimated using NHS Staff Earnings Estimates and added to the annuitised device costs as appropriate. Wider resource use incurred by the NHS, social services, families and employers will be collected using an adapted version of the Client Service Receipt Inventory (CSRI) [20]. The CSRI is completed through interviews with the participant collected at baseline, 4, 8 and 16 weeks follow-up. These will be costed using published unit costs, e.g. from the Personal Social Services Research Unit (PSSRU), NHS reference costs and British National Formulary. The outcome measure used for the economic analysis will be quality-adjusted life years (QALYs), derived from utilities captured using the EuroQoL EQ-5D-5L instrument, which is responsive in depression even over short time periods [28], and valued using the recommended tariff at the time of analysis [29]. Following the NICE recommendations, utility values in reference case analyses will be calculated by mapping the 5L descriptive system data onto the 3L value set [29]. Using this information on costs and benefits, an incremental cost-utility analysis will be conducted and reported using an accepted methodology, including a cost-effectiveness acceptability curve showing the probability that the intervention is cost-effective at a range of threshold values for the willingness to pay per QALY [30].

### Interim analyses {21b}

No interim analyses are planned.

### Methods for additional analyses (e.g. subgroup analyses) {20b}

No additional analyses are planned.

### Methods in analysis to handle protocol non-adherence and any statistical methods to handle missing data {20c}

Missing values in all outcomes will be checked and reported across treatment groups and follow-up time. As the outcome will be repeatedly measured, a two-level logistic regression with patients as a level 2 unit will be performed to test the influence of treatment status and baseline measures on outcome missingness. The missing value patterns and the results from multilevel logistic regression modelling will be used to inform missing value imputation under missing rt Random (MAR) assumption [31]. Although multilevel modelling for repeated measures could be automatically taken into account, missing outcomes under MAR assumption may be used to give sensible results [32]. To ensure all randomised participants will be included in the analysis, the missing values will be imputed using multilevel modelling to quantify the treatment effect estimates [32]. The results of modelling on observed data will be used as a sensitivity analysis to check the robustness of results sensitive to missing values. The STATA 17 and REALCOM-IMPUTE software will be used to impute missing values by means of Markov chain Monte Carlo (MCMC) approach for multilevel data [32].

### Plans to give access to the full protocol, participant-level data and statistical code {31c}

This document is the full protocol. Anyone interested in participant-level data or statistical code should contact the corresponding author.

### Oversight and monitoring

#### Composition of the coordinating centre and trial steering committee {5d}

The CI has overall responsibility for the study and shall oversee all study management, with oversight from the rest of the research team. The data custodian will be the CI. The coordinating site is staff employed by the University of Nottingham. The trial management group is responsible for conducting the trial and will meet fortnightly to discuss trial progress. This will comprise of the CI, study researchers, programme manager and study statistician.

The trial will be overseen by an independent ARC EM Scientific Committee. The members of the committee will be drawn externally from outside the institutions that the research team currently work to ensure its independence of the research team. It will serve the function of a Trial Steering Committee (TSC) and a Data Monitoring Committee (DMC). The trial will be reviewed every 6 months through reports to this committee and presentations of progress to the committee by the study team.

#### Composition of the data monitoring committee, its role and reporting structure {21a}

The data monitoring committee will review the interim safety data and periodically review the conduct of the study. The steering committee takes responsibility for the scientific validity of the study protocol, assessment of study quality and conduct and for the scientific quality of the final study report. The sponsor and the investigators participating in the trial bear the final responsibility for the conduct of the trial.

#### Adverse event reporting and harms {22}

All adverse events (AEs) and adverse device effects (ADEs) will be assessed for seriousness, expectedness and causality. An AE whose causal relationship to the study device is assessed by the CI as “possible”, “probable” or “definite” will be reported as an adverse device effect (ADE). Side effects will be closely monitored and reported in accordance with the procedures outlined in this protocol and by the research team through the adverse events form. All AEs, SAEs, ADEs and SADEs will be documented in the participant’s records and CRFs. Participants will be asked to contact the study site immediately in the event of any adverse events. The CI shall be informed immediately of any adverse events and shall determine seriousness and relationship in conjunction with any treating medical practitioners. The manufacturer and supplier will have the responsibility for safety reporting to the Medicines and Healthcare Products Regulatory Agency (MHRA) via usual device post-market vigilance arrangements. Any participant who experiences an adverse event may be withdrawn from the study at the discretion of the CI.

#### Frequency and plans for auditing trial conduct {23}

For quality assurance, the sponsor, the ethics committee or an independent intern study monitor may visit the research site. Direct access to the source data and all study-related files will be granted on such occasions. All involved parties will be required to keep the participant data strictly confidential.

#### Plans for communicating important protocol amendments to relevant parties (e.g. trial participants, ethical committees) {25}

This document is the full protocol. Anyone interested in other data or documentation should contact the corresponding author. Changes to the study protocol and relevant study documents will be submitted to the ethics committee for approval before implementation.

Any changes to the protocol will include notifying the sponsor and funder first. The CI will then notify the sites, and copy of the revised protocol will be sent to the sites to add to the Investigator Site File. Any deviations from the protocol will be fully documented using a breach report form. Any updates to the protocol will also be made on the clinical trial registry https://www.isrctn.com/ISRCTN11853110.

#### Dissemination plans {31a}

Participants in the trial will receive a summary of the findings. In addition, we will present the findings at national and international conferences.

#### Patient and public involvement and engagement

In developing this trial, we have consulted with a number of Patient and Public Involvement and Engagement (PPI/E) representatives, and the intervention was co-designed through PPI/E input. PPI/E representatives have been integral in shaping the design of the study, supporting documents and the design of the recruitment strategy. They will continue to be involved throughout the study and in the interpretation and dissemination of findings. Involvement in this trial is supported and facilitated by PPI/E representatives Rebecca McNaughton, Debbie Butler and Fred Higton. The PPI/E representatives have provided input on all our patient-facing documents, including the PIS, and consent forms and have shared feedback on using the Alpha-Stim AID device. Refinements and alterations to documents were made based on their suggestions. PPI/E representatives have also reviewed our outcome measures to ensure acceptability. We collaborated with PPI/E representatives on the development of the instructional video which was produced in light of the COVID-19 pandemic and to reduce face-to-face contact. Our PPI/E panel will play a pivotal role in the dissemination of findings and will be involved in the interpretation of results. Their perspectives will form an essential part of all analysis; without their input, such analysis will be regarded as incomplete.

## Discussion

Although the evidence base, tolerability, patient preference and ease of use are promising, there is a need for a further RCT of the long-term clinical and cost-effectiveness in treatment-seeking patients with moderate depression in primary care [18]. Alpha-Stim AID is rarely used in NHS care in the UK, but it is used privately. Thus, there is a growing issue of inequity, as depression disproportionately affect the poorest in society, yet Alpha-Stim AID is primarily only available to people who can afford to purchase it but not to the majority of people who cannot afford it. During the early stages of project development, PPI/E representatives from NIHR MindTech MedTech Co-operative and a PPI/E representative from NIHR ARC East Midlands expressed that restricted options for interventions (antidepressants and psychological treatment as the only options) may deter people with depression from getting help. This is corroborated by previous research [33]. PPI/E representatives expressed that they would get the help they needed if there were accessible alternative safe, efficacious, cost-effective, easy to use NHS treatments.

The Alpha-Stim AID device has the potential to increase choice which in turn could mean improving access. Many people have negative thoughts of antidepressants and may also have negative experiences of psychotherapy. Therefore, they deny themselves treatment. Given the high volume of patients with depression [27] but the high rate of spontaneous remission and responsiveness to first-line antidepressant treatments, we think the likely place for Alpha-Stim AID would be the primary care clinical pathway. It could be for patients with moderate to severe depression who have not responded to a first-line antidepressant given at a therapeutic dose for 6 weeks or more or those who have been offered an antidepressant but are unwilling to take it, or unable to tolerate it. Such patients are unlikely to remit, justifying the use of an alternative treatment approach such as Alpha-Stim AID should it prove clinically effective in this research. It could also be a more favourable treatment option for patients from socially disadvantaged backgrounds, who may not wish to or may be less readily able to access psychological therapy. Alpha-Stim AID is now starting to become established for the treatment of anxiety through direct sale to the public but it is also CE marked for direct sale to the public for depression with evidence only of effectiveness against depression symptoms in people with primary anxiety disorders. There is an urgent need to address depression in young people and adults because of the increased presentation of patients with depression in primary care, reflected in the rising use of antidepressants to 71 million prescriptions in 2018 in England [34] and the rising demand for talking therapies but also evidence of a rising demand for other effective alternatives. Alpha-Stim AID meets policy priorities for offering choice for the treatment of depression at a cost similar to antidepressants and at a lower cost than psychological treatment. However, most people who might benefit from this treatment cannot afford to pay for it. Hence, further research is required to explore its long-term clinical and cost-effectiveness for managing depression in NHS primary care.

Following on from a successful RCT conducted before the COVID-19 pandemic by our research team using remote assessment, consent, treatment delivery and follow-up [35], we had already planned to use these methods in addition to face-to-face contact before the onset of the pandemic which severely restricted face-to-face contact with patients for non-essential care, including a period of 6 months when the recruitment to this study was delayed. With minimal changes to our protocol (delivery of the device directly to the patient’s home and an instructional video rather than the GP practice providing delivery and instructions), we have been able to successfully deliver this trial despite a national lockdown on movement throughout most of the study period. Participants are now also familiar with videoconferencing in their private lives and as a means of consulting about their health. Therefore, when feasible and allowed by regulatory bodies, we will continue to use remote methods for many of our trials regardless of pandemic restrictions.

## Trial status

The trial is currently actively recruiting via GP surgeries. The first 150 participants were randomised to the trial as of 27 September 2021. Recruitment is due to end early 2022. The trial was registered on ISRTCN Registry (ISRCTN11853110 10.1186/ISRCTN11853110) in August 2020. If significant changes requiring the modification of this protocol are required, the changes will be submitted for approval to the HRA and REC.

## Abbreviations

ADE: Adverse device effect
ARC: Applied Research Collaboration
CES: Cranial electrotherapy stimulation
RCT: Randomised controlled trial
CRN: Clinical Research Network
PPI/E: Patient and Public Involvement and Engagement
SADE: Serious adverse device event
SAE: Serious adverse event
